# Erratum

**DOI:** 10.4269/ajtmh.17-0050err

**Published:** 2018-02

In *Am J Trop Med Hyg 97(3):* 681–683 (https://doi.org/10.4269/ajtmh.17-0050) by Barda and others, there were a few errors in the text.

The second to last sentence of the first paragraph should read “However, ivermectin is often not easily available in *S. stercoralis* endemic areas, outside preventive chemotherapy (PC) programs targeting lymphatic filariasis (LF) or onchocercosis.”

The second paragraph should read “The potential benefit of PC with ivermectin in patients infected with *S. stercoralis* has been observed previously by a few studies that were reporting the results of control programs aiming at the elimination of onchocercosis.”

The third sentence in the third paragraph should read “Zanzibar, in addition to LF, is also endemic for infections with STH and *Schistosoma hematobium* and PC either school-based and/or community-based has been ongoing as part of the Zanzibar National Helminth Control Program and LF Elimination Program since 1995.”

The seventh sentence in the third paragraph should read “In each of the six treatment rounds (1995–2014) (Figure 1), ivermectin was administered together with albendazole as a single dose of 200 µg/kg.

Reference 1 should be added at the end of the third paragraph.

Email addresses for Beatrice Barda and Jennifer Keiser should be Beatrice.barda@swisstph.ch and Jennifer.keiser@swisstph.ch.

